# Determining Hopelessness Levels and Related Factors in Veterinary Students

**DOI:** 10.3390/bs13100798

**Published:** 2023-09-26

**Authors:** Erhan Yüksel, Özlem Yüksel

**Affiliations:** Department of Veterinary History and Deontology, Veterinary Faculty, Sivas Cumhuriyet University, Sivas 58140, Turkey; ozlemyuksel@cumhuriyet.edu.tr

**Keywords:** causative factors, hopelessness level, veterinary students

## Abstract

This study addresses the level of hopelessness experienced by last-year students at a faculty of veterinary medicine. Moreover, it identifies the factors behind this emotion. A face-to-face questionnaire was administered to 238 last-year students from 2017 to 2021. The questionnaire included questions about sociodemographic and other characteristics, along with the Beck Hopelessness Scale. Data analysis included descriptive statistics, factor analysis, and chi-square tests. The findings suggested that nearly 60% of the respondents experienced hopelessness. The analysis also revealed a significant relation between hopelessness levels and variables such as year, gender, expected time of first employment, and psychological status. The results suggested that the current state of mental health is worrying for the veterinary faculty students and therefore for the future veterinarians. For this reason, it can be argued that steps to be taken towards a solution in the veterinary education periods are required.

## 1. Introduction

Defined as a state of uneasiness or fear [1], anxiety refers to individuals’ unpleasant emotional reactions to stressful situations in the form of worry and agitation [2]. An anxious person is in fear and feels uncomfortable [3]. Hopelessness, which is used interchangeably with the concept of anxiety and depression, is characterized by pessimistic thoughts about life and the future, the feeling that one will not experience an improvement in one’s condition [4,5,6,7], and negative expectations for the future [8]. Numerous negativities faced by individuals throughout life cause individuals to feel anxious about their lives, to have a more negative outlook on life, and to foster a sense of hopelessness [9,10]. According to studies on clinical groups associated with despair, which is critical for an individual’s psychological condition, hopelessness is substantially correlated with depression, future suicidal urge, and schizophrenia. The level of hopelessness, which is one of the parameters used in determining mental health, is also accepted as an important indicator of public health [11,12,13].

It was reported that during university education, one of the defining periods of life, students are affected by numerous factors exacerbating their levels of anxiety. Many studies have reported that in the last year of university education, students’ anxiety is exacerbated owing to graduation stress, fear of unemployment, and concerns related to future profession [14,15,16]. Due to the increasing concerns about the mental health of future generations, studies stress the need to investigate numerous aspects impacting the mental health of students during their university years and eliminating the effective ones based on the findings [17,18,19].

The educational adventure in veterinary faculties includes a very difficult and intense process. The requirements of the education program, the expected degree of achievement, and other personal factors affect the mental health of veterinary students [20,21,22]. According to the results of research conducted on veterinary faculty students, they experience higher levels of psychological problems compared to the general population and students from other vocational programs. It is highlighted that this circumstance is a concerning issue [20,21,23,24,25]. Furthermore, it is claimed that this unfavorable picture of veterinary faculty students’ mental health persists in their professional lives as veterinarians after graduation [20,21].

Veterinarians, who are among medical professionals, admittedly work under difficult conditions, and these conditions are regarded as the possible reasons behind the concerns related to veterinarians’ mental health, which are frequently addressed in the literature in light of data. Recent studies have revealed that compared with other healthcare professionals, veterinarians face disproportionately higher suicide rates and that veterinary school students exhibit higher levels of discomposure, depression, and symptoms of anxiety disorder [26,27,28,29,30,31,32]. It is declared that in order to provide timely support services to vulnerable groups, it is important to establish the mental health status of students as well as to illuminate the factors associated with poor mental health status [22]. As such, it has become imperative to investigate the role of veterinary medicine education in students’ mental problems in light of the factors that predict the high levels of depression and anxiety they experience, such as homesickness, issues related to physical health, and the challenging curriculum, which have recently been added to by the impact of the COVID-19 pandemic [28,29,30,32]. Although a study conducted in Turkey in 2012 addressed the hopelessness levels of veterinary physicians and veterinary students through Beck’s Hopelessness Scale (BHS) [33], there is currently no study that has been carried out in veterinarians and veterinary students that has examined anxiety, hopelessness levels, and influencing factors.

In this study, it was aimed to determine the hopelessness level, which is an indicator of mental health, of the final year students of a faculty of veterinary medicine, for which there is no up-to-date data for Turkey, with a reliable method, and to determine the factors behind the feeling of hopelessness, keeping in mind their relations with the next-generation veterinarian population.

## 2. Materials and Methods

This study was approved by the Sivas Cumhuriyet University Scientific Research and Publication Ethics Social and Human Sciences Committee (approval no: 2017/040). The population of the study comprised 238 students from a faculty of veterinary medicine located in the Central Anatolia Region in Turkey. Between 2017 and 2021, face-to-face interviews were held with last-year veterinary students undertaking internships. Furthermore, a questionnaire was administered to the students. The students were included in the study on a voluntary basis, and informed consent was obtained from each student before the research. No clinical screening was performed for the mental health of the participants, and there is no information about whether a mental health screening was performed before this study or possible treatment processes. “BHS” [34], which is a reliable and easy-to-apply method that is frequently preferred in studies, was used to determine the hopelessness levels of the participants [8,12,35,36]. The scale was adapted to Turkish by Seber et al., and its validity and reliability were evaluated by Seber et al. [37] and Durak and Palabıyıkoğlu [38]. The 20 items in the BHS were assigned point values as follows: Yes (positive answer) = 1; No (negative answer) = 0. The arithmetic sum of item responses revealed the “Hopelessness score”, reflecting the level of discomposure experienced by the respondent. In line with the literature, scores ranging between 0 and 3 were considered within the normal range, scores from 4 to 8 manifested mild hopelessness, scores from 9 to 14 indicated moderate hopelessness (requiring frequent follow-up), and scores greater than 14 identified severe hopelessness (definitely suicidal) [37,38].

In addition to the BHS, questions regarding the determination of sociodemographic and other characteristics that were used in previous studies [20,21,23] on the subject and thought to be related to the level of hopelessness by the research team were included in the questionnaire form in order to define the characteristics of the research population. 

In the first stage of the study, descriptive statistics regarding the sociodemographic and other characteristics of the participants were collected. A comprehensive EFA, including a scatterplot, factor loadings, and communalities (h2), was not performed for BHS, whose safety and validity have already been proven many times before by previous studies. The EFA included the Kaiser–Meyer–Olkin (KMO) test to measure sample adequacy only, and if the obtained correlation matrix was the unit matrix where all diagonal terms are 1 and non-diagonal terms are 0, Bartlett’s test and Cronbach’s alpha, which is the reliability coefficient, were used. In addition, the Cronbach’s alpha value, the single-factor structure, was calculated for the scale consisting of 20 questions. In the study, a first-level confirmatory factor analysis model was created and the factors in the BHS structure were tested. The goodness of fit of the model was tested and the CFI and RMSEA values were calculated. In the last stage, the “BHS” scores of the participants were evaluated vis-à-vis their sociodemographic and other characteristics through the chi-square test. Spss 21 and Amos 21 software were used for the statistical analysis.

## 3. Results

The sociodemographic data indicated that, of the participants, 70% were male, four-fifths were under the age of 25, and almost all were single. In addition, of the participants, more than four-fifths stated that they lived in a student house or dormitory, more than two-thirds were from the low- and middle-income groups, more than half planned to have their own clinical practice after graduation, nearly 40% expected to start gaining professional experience upon graduation, almost two-thirds believed that they would be able to find a job within 6 months, more than 70% described their psychological status as poor or very poor, and more than half decided to become veterinary students themselves. Table 1 displays the frequency distribution of the data on sociodemographic and other characteristics of the participants.

The BHS, the reliability and validity of which had already been established and the factor structure of which had been identified, was used in the study for data collection, and the accuracy of its factor structure was tested through confirmatory factor analysis. In the first step of the exploratory factor analysis, the Kaiser–Meyer–Olkin (KMO) test was performed to measure the sampling adequacy, and the measurement value was 0.881 (Table 2). The fact that the measurement value was close to 1 indicated that the available data groups were suitable for exploratory factor analysis. In addition, Bartlett’s test was performed to determine whether the correlation matrix obtained in the analysis was a unit matrix in which all diagonal terms were 1 and nondiagonal terms were 0. The null hypothesis suggesting that the correlation matrix of the scale was zero was rejected (Bartlett’s test *p* < 0.001). The results of the two tests indicated that the sample was suitable for exploratory factor analysis (Table 2).

Since the literature review revealed that the scale had a single-factor structure [36,39,40,41], the number of factors was determined as 1. The total variance explanation of these factors was 29.975%. Furthermore, the Cronbach’s alpha value of the 20-item scale, which denotes the reliability coefficient of the one-factor structure, was calculated to be 0.864. The factors in the structure of the BHS were tested by creating a first-level confirmatory factor analysis model. In the BHS, comprising a single dimension, 20 observed variables were represented by 20 rectangles. In the path diagram drawn with the help of the AMOS, all standardized values obtained were below 1. When the parameter values for the BHS items were examined in the track diagram, the ratio of degrees of freedom (170) for the goodness-of-fit χ^2^ value (336.381), where the model was evaluated as a whole, was calculated as 1.979. This value was below 2, indicating that the model was a good fit. Similarly, the CFI (0.847) and RMSEA values (0.064) also indicated a good fit (Table 3).

**Figure 1:**
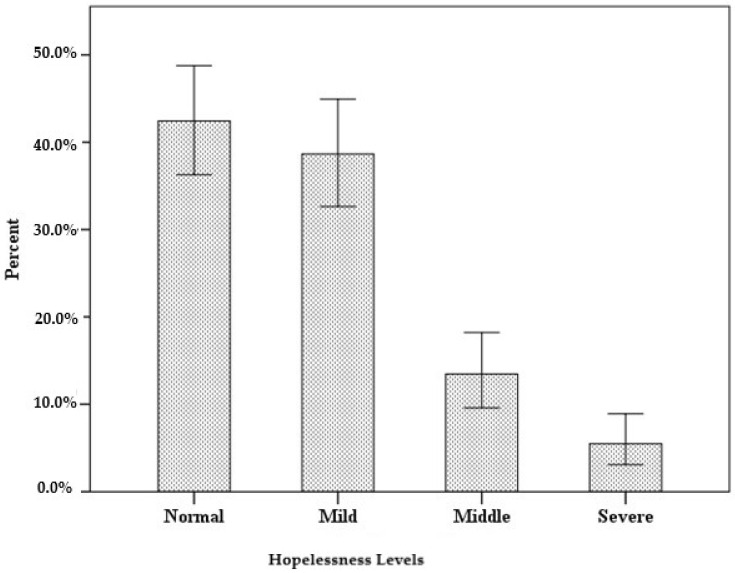
The distribution of the participants’ levels of hopelessness.

The scores obtained by participants from the BHS, which were found to be appropriate through the explanatory factor analysis, revealed that nearly three-fifths of the participants had mild, moderate, or severe levels of hopelessness and therefore had anxiety. The distribution of the participants’ levels of hopelessness is presented in Table 4 and Figure 1. 

Data on the distribution of participants’ hopelessness levels by scale scores and statistical evaluation of this distribution vis-à-vis the identified variables are presented in Table 5. Accordingly, males were observed to be more hopeless than females, those aged 26 and over were more hopeless than those aged 25 or less, and those with middle- and high-income levels were more hopeless than those from low-income levels. No statistically significant difference was observed between these parameters and hopelessness scores (*p* > 0.05) (Table 5). However, the participants in the university during 2020 were more hopeless than those from other years, those who expected to find employment later were more hopeless than the participants who expected to find a job earlier, and those who defined their psychological status as bad or very bad were more hopeless than those who selected the other answers. A statistically significant relation was observed between the hopelessness scores and the year when the questionnaire was answered, the estimated period before employment upon graduation, and psychological status (*p* < 0.05) (Table 5). In addition, those who chose the faculty for money, planned to work in the public sector, and expected to have a good income after graduation were more hopeless than those who chose the other answers in the respective items. However, the relation between these parameters and the hopelessness scores was not statistically significant (*p* > 0.05) (Table 5).

## 4. Discussion

The study carried out with veterinary faculty students concluded that nearly three-fifths of the participants had mild, middle, or severe levels of hopelessness (57.6%) (Table 4). Furthermore, the rate of participants with normal, mild, or severe levels of hopelessness statistically significantly increased each consecutive year when the questionnaire was administered (*p* = 0.003). With regard to hopelessness, regarded as an indicator of individuals’ mental health status [42], a study conducted in the UK revealed that the suicide rate among veterinarians was two times higher than among other healthcare professionals and four times higher than among the general population [30]. Similarly, studies carried out in the USA [27], Australia [29], and Norway [43] indicated a higher suicide rate among veterinarians than among the general population. Similar studies comparing veterinary medicine with other professions have found that veterinarians have a higher risk of depression and suicide than other professions in several countries (e.g., the UK, the USA, Australia, and Austria), and this situation has been reported as worrying [44,45,46,47]. The reasons for this situation were concluded to be the difficult working conditions, the level of responsibility the veterinary medicine profession commands, and the curriculum, in addition to personal issues. However, 32% of first-year veterinary students exhibited symptoms of clinical depression. Veterinary students experienced higher levels of depression, anxiety, and discomposure compared with medical students and the general population, resulting from the challenging curriculum of the faculty and the pressure to succeed [28]. A recent study concluded that the prevalence of depression among veterinary students in Germany was 45.9% and that the suicidal tendency was 19.9%. These rates were approximately 10 times higher than those among the general population [25]. However, in a study conducted in the USA in 2019, 22.6% of veterinary students had depression and 52.3% had general anxiety [20]. In 2023, it was concluded that 55.3% of veterinary students in Austria showed moderate depressive symptoms and that 52.6% showed moderate anxiety symptoms [22]. Another study emphasized that the COVID-19 pandemic, which had impacts stretching all over the world starting in 2020, should be considered among the factors negatively affecting the mental state of veterinary students [32,48]. It can be hypothesized that the literature findings on veterinary students’ levels of discomposure, anxiety, and depression may suggest that veterinary students are at risk of experiencing hopelessness, and this study investigates the levels of hopelessness experienced by the participants. Moreover, it may be claimed that since the findings of this study predict an increase in the suicidal tendency among veterinarians, the experiences of the veterinary students should be the point of focus in determining the precautions to be taken against concerns regarding the mental state of veterinarians [31]. In addition, the fact that normal, mild, and severe levels of hopelessness were more prevalent among participants who answered the questionnaire in 2020 can be attributed to the onset of the COVID-19 pandemic and its grave impact on Turkey and around the world [49].

In light of the fact that, according to the Turkish Statistical Institute, the suicide rate per 100,000 was 4.94 in Turkey in 2021 [50], this study’s finding that at least 7 out of every 10 participants (71.5%) (Table 1) in the population described their mental status as bad or very bad is fairly striking. Indeed, other relevant studies in the literature support this remarkable finding on veterinary students’ mental status [25,28,31,48]. In addition, considering the statistical significance of the correlation between psychological status and hopelessness levels (*p* = 0.012), it is clear that psychological status is important and naturally one of the factors contributing to hopelessness.

Demographic data revealed that between 2017 and 2021, nearly three-quarters of the final-year veterinary students were male (Table 1). Similarly, other Turkish studies in the literature carried out on different dates found that the number of male veterinary faculty students was higher than that of female students [51,52,53]. Accordingly, it is possible to claim that this study’s finding on gender distribution in the veterinary faculty of the university where the study was conducted is parallel to the findings in the literature. As Başağaç Gül et al. [54] emphasized in their study, this may be due to the fact that veterinary medicine is regarded as a male-oriented profession in Turkey, despite the increase in the number of women in the field over the years. Moreover, despite the absence of a statistically significant relation between the levels of hopelessness and gender, female participants were found to exhibit higher levels of hopelessness (66.2%) compared with male participants (53.7%) (Table 5). This can be attributed to the fact that women in Turkish society are subjected to anxiety-provoking experiences more than their counterparts due to their perceived place in social life and maternal characteristics [55], which was emphasized in a recent study.

A study conducted with veterinarians in 2012 on the length of the period until employment after graduation reported that nearly 60% of the participants were able to find permanent employment within six months of their graduation [56]. Considering that 65.1% of the participants in this study expected to find employment within six months after graduation (Table 1), the participants’ expectations regarding the period of time required to find employment are realistic. In addition, more than 60% of the participants who believed that it would take them longer than six months after graduation to find a job were found to have normal, mild, and severe levels of hopelessness. This indicated a significant relation between hopelessness levels and expected period of time before finding permanent employment after graduation (Table 5). These findings suggest that despite the participants’ optimistic outlook regarding the duration of finding a job after graduation, this factor still pushes veterinary students into hopelessness. Relevant to the expectations regarding employment after graduation, of the participants, 51.3% aimed to have their own clinical practice and 9.7% wished to work in the private sector. This implied that more than three-fifths of the participants planned to work outside of the public sector (Table 1). This finding is in line with the finding of Özen et al. [56] that veterinarians find the private sector more appealing than the public sector. In light of this finding, despite the lack of a statistically significant relation between the participants’ levels of hopelessness and the preferred field of employment (*p* = 0.441) (Table 5), the fact that those who plan to work in the public sector make up the majority among those who exhibit normal, mild, and severe hopelessness (61.7%) (Table 5) may be related to the limited employment capacity in the public sector in Turkey [57].

In terms of the participants’ expectations from the department, the most frequent answer provided to the relevant item was working in the field interested in/in a good job (Table 1). In addition, more than half of the participants selected the own will option for the reason for choosing this faculty item, indicating that they had made a conscious choice to become prospective veterinarians (Table 1). These two findings differ from those obtained by Özen et al. in 2012 [33] suggesting that a significant percentage of participants had not performed an informed and conscious decision-making process while choosing their career paths and were worried that they would not be able to work in the fields they wanted. Nevertheless, it is possible to suggest that the prediction of Özen et al. that conscious preference for faculties of veterinary medicine would be more prevalent in the coming years, which would reduce the number of veterinary students with career concerns [33], has started to come true. Indeed, no statistically significant relation was detected between the answers provided to the relevant items; thus, it can be inferred that the number of students who chose the faculty of veterinary medicine after an informed decision-making process increased, which translated into a compatibility between veterinary students’ academic field and intended profession.

Without detriment to the importance of the findings, the study’s limitations are that the study was cross-sectional and that the participants only comprised students from a faculty of veterinary medicine located in the Central Anatolia Region. It can be argued that to obtain more reliable data on the mental status of Turkish veterinary students, the veterinarians of the future, there is a need for studies with a population representing students from all years of all veterinary faculties in Turkey.

## 5. Conclusions

In conclusion, this study found that final-year students at a faculty of veterinary medicine experienced varying levels of hopelessness, regarded as an indicator of mental health. As a result, conditions such as a decrease in academic achievement levels, the development of unhealthy habits, depression, and anxiety can occur among veterinary faculty students. There were significant relations between the participants’ hopelessness levels and the variables of year, gender, estimated time of finding employment after graduation, and psychological status. With the findings of the present study, it is possible to state that an updated contribution to the literature on the mental health of veterinary students in Turkey has been made and that the data can be used to suggest precautions to be taken for the mental health of both veterinary faculty students and future veterinarians. The findings of this study are significant, as they can inform the measures to be taken by faculties against this bleak outlook for the sake of future veterinarians. Further, in light of the findings of this study, it is imperative to carry out a Turkey-wide study where the levels of hopelessness experienced by veterinary students and all possible parameters exacerbating this sentiment are addressed and a roadmap for the elimination of this issue is proposed. It can be argued that, in addition to the need for more comprehensive research on this subject, which is a concern for future veterinarians, it will be beneficial to apply regular clinical screening tests for the mental health of veterinary students during the education process, and it is important to establish units that will guide students in need of treatment.

## Figures and Tables

**Table 1:** Frequency distribution of the data on sociodemographic and other characteristics of the participants.

Variables	Categories	*n*	%
Gender	Female	74	31.1
	Male	164	68.9
Age	<25 ≤25	195	81.9
	>26 ≥26	43	18.1
Marital Status	Single	220	92.4
	Engaged	11	4.6
	Married	7	2.9
Year	2017	44	18.5
	2018	51	21.4
	2019	50	21
	2020	51	21.4
	2021	37	15.5
Place of Residence	Metropolitan city	85	35.7
	Urban setting	60	25.2
	Urban	54	22.7
	Town	13	5.5
	Village	26	10.9
Mother’s Occupation	Unemployed	191	80.3
	Wage earner	27	11.3
	Retired	10	4.2
	Freelancer	10	4.2
Father’s Occupation	Retired—unemployed	70	29.4
	Farmer	37	15.5
	Freelancer	49	20.6
	Wage earner	81	34
Number of Siblings	≤2	102	42.9
	>2	135	56.7
Place of Residence during Study	Family house	45	18.9
	Dormitory	83	34.9
	Student house	110	46.2
Income Levels	Low	60	25.2
	Middle	124	52.1
	High	52	22.7
Career Preference upon Graduation	Own clinical practice	122	51.3
	Public sector	60	25.2
	Private sector	23	9.7
	Academic career	20	8.4
	Undecided	10	4.2
Expectations from the Department	Learning a profession	89	37.4
	Working in the field interested in/in a good job	81	34
	Having status	54	22.7
	A good and happy life	7	2.9
	Having a good income	3	1.3
Estimated Period before Employment upon Graduation	0–6 months	155	65.1
	6 months–1 year	39	16.4
	1–2 years	36	15.1
	2 years and above	8	3.4
Psychological Status (Fifth Year)	Very good	13	5.5
	Good	44	18.5
	Bad	97	40.8
	Very bad	73	30.7
Reason for Choosing this Faculty	Own will	133	55.9
	Money	28	11.8
	External factors	52	21.8

*n*: frequency; %: percentage.

**Table 2:** KMO and Bartlett’s test.

Kaiser–Meyer–Olkin Measurement for Sampling Adequacy		0.881
Bartlett’s Test	Chi-square	1212.49
	df	190
	p	<0.001

**Table 3:** Fit indices for confirmatory factor analysis.

Χ^2^	Df	*p*	Χ^2^/df	CFI	RMSEA
336.381	170	<0.001	1.979	0.847	0.064

**Table 4:** Classification of participants’ levels of hopelessness by BHS scores.

Hopelessness Levels	*n*	%	BHS Score		
			Min	Max	Mean ± Std. Deviation
Normal	101	42.4	0	3	1.68 ± 0.98
Mild	92	38.7	4	8	5.62 ± 1.33
Middle	32	13.4	9	14	11.25 ± 1.83
Severe	13	5.5	15	20	16.69 ± 1.60

*n*: frequency; %: percentage, BHS: Beck’s Hopelessness Scale.

**Table 5:** Evaluation of the participants’ Beck Hopelessness Scale scores vis-à-vis their sociodemographic characteristics.

Variables	Categories	Hopelessness Level *n* (%)				*p* Value
		Normal	Mild	Middle	Severe	
Gender	Female	25 (33.8)	32 (43.2)	10 (13.5)	7 (9.5)	0.132
	Male	76 (46.3)	60 (36.6)	22 (13.4)	6 (3.7)	
Age	≤25	86 (44.1)	75 (38.5)	23 (11.8)	11 (5.6)	0.397
	≥26	15 (34.9)	17 (39.5)	9 (20.9)	2 (4.7)	
Income Level	Low	23 (38.3)	25 (41.7)	7 (11.7)	5 (8.3)	0.887
	Middle	54 (43.5)	48 (38.7)	17 (13.7)	5 (4)	
	High	23 (44.2)	18 (34.6)	8 (15.4)	3 (5.8)	
Year	2017	23 (52.3)	17 (38.6)	4 (9.1)	0 (0)	**0.003**
	2018	22 (43.1)	23 (45.1)	6 (11.8)	0 (0)	
	2019	20 (40)	22 (44)	4 (8)	4 (8)	
	2020	18 (35.3)	16 (31.4)	12 (23.5)	5 (9.8)	
	2021	14(37.8)	14 (37.8)	5 (135.5)	4 (10.8)	
Career Preference upon Graduation	Own clinical practice	53 (43.4)	48 (39.3)	15 (12.3)	6 (4.9)	0.441
	Public sector	23 (38.3)	23 (38.3)	10 (16.7)	4 (6.7)	
	Private sector	12 (52.2)	6 (26.1)	4 (17.4)	1 (4.3)	
	Academic career	9 (45.0)	10 (50.0)	0 (0)	1 (10.0)	
	Undecided	4 (40.0)	2 (37.9)	3 (13.6)	1 (5.5)	
Expectations from the Department	Learning a profession	30 (33.7)	41 (46.1)	12 (13.5)	6 (6.7)	0.108
	Working in the field interested in/in a good job	33 (40.7)	29 (35.8)	13 (16.0)	6 (6.7)	
	Having status	32 (59.3)	15 (27.8)	6 (11.1)	1 (1.9)	
	A good and happy life	4 (57.1)	3 (42.9)	0 (0)	0 (0)	
	Having a good income	0 (0)	2 (66.7)	1 (33.3)	0 (0)	
Estimated Period before Employment upon Graduation	0–6 months	83 (53.5)	53 (34.2)	12 (7.7)	7 (4.5)	**<0.001**
	6 months–1 year	12 (30.8)	17 (43.6)	10 (25.6)	0 (0)	
	1–2 years	5 (13.9)	19 (52.8)	9 (25.0)	3 (8.3)	
	2 years and above	1 (12.5)	3 (37.5)	1 (12.5)	3 (37.5)	
Psychological Status (5th-year)	Very good	8 (61.5)	4 (30.8)	1 (7.7)	0 (0)	**0.012**
	Good	29 (65.9)	12 (27.3)	2 (4.5)	1 (2.3)	
	Bad	32 (33.0)	44 (45.4)	17 (17.5)	4 (4.1)	
	Very bad	28 (38.4)	27 (37.0)	11 (15.1)	7 (9.6)	
Reason for Choosing this Faculty	Own will	54 (40.6)	55 (41.4)	17 (12.8)	7 (5.3)	0.610
	Money	8 (28.6)	10 (35.7)	7 (25.0)	3 (10.7)	
	External factors	20 (38.5)	21 (40.4)	8 (15.4)	3 (5.8)	

*n*: frequency; %: percentage.; *p* values given in bold representing the statistically significance.

## Data Availability

The data that support the findings of this study are not publicly available due to privacy or ethical restrictions.
